# Predicting the Need for Advanced Respiratory Support in COVID-19 Patients During the Initial Pandemic Phase: A Retrospective Analysis

**DOI:** 10.7759/cureus.64678

**Published:** 2024-07-16

**Authors:** Tuğba Çiçek, Melahat Uzel Sener, Ayperi Öztürk

**Affiliations:** 1 Chest Disease, Konya Numune Hospital, Konya, TUR; 2 Chest Disease, Health Science University, Ataturk Chest Diseases and Thoracic Surgery Training and Research Hospital, Ankara, TUR

**Keywords:** c-reactive proteins (crp), age, serum ferritin, systemic immune-inflammation response index, predictors, covid-19, advanced respiratory support

## Abstract

Background: The coronavirus disease 2019 (COVID-19) pandemic, caused by severe acute respiratory syndrome coronavirus 2 (SARS-CoV-2), led to high morbidity and mortality rates worldwide. It is known that some patients, initially hospitalized in general wards, deteriorate over time and require advanced respiratory support (ARS). This study aimed to identify key risk factors predicting the need for ARS in patients during the pandemic's early months.

Methodology: In this retrospective study, we included patients admitted within the first three months of the pandemic who were diagnosed with COVID-19 via reverse transcription polymerase chain reaction (RT-PCR). The patients who required ARS or invasive mechanical ventilation at admission were excluded. Data on demographics, comorbidities, symptoms, vital signs, and laboratory parameters were collected. Statistical analyses, including multivariate logistic regression and receiver operating characteristic (ROC) curve analysis, were performed to identify independent predictors of ARS and determine the cut-off point.

Results: Among 162 patients, 32.1% required ARS. Key differences between ARS and non-ARS groups included age, body mass index (BMI), coronary artery disease prevalence, neutrophil count, C-reactive protein (CRP), ferritin, D-dimer, troponin T levels, neutrophil-to-lymphocyte ratio (NLR), systemic immune-inflammation response index (SIRI), and symptom-to-admission time. Multivariate analysis revealed that age, elevated CRP levels, elevated ferritin levels, and SIRI were significant predictors for ARS. The ROC curve for SIRI showed an area under the curve (AUC) of 0.785, with a cut-off value of 1.915.

Conclusions: Age, CRP levels, ferritin levels, and SIRI are crucial predictors of the need for ARS in COVID-19 patients. The early identification of high-risk patients is essential for timely interventions and resource optimization, particularly during the early stages of pandemics. These insights may assist in optimizing strategies for future respiratory health crisis management.

## Introduction

Coronavirus disease 2019 (COVID-19), a highly contagious viral infection caused by severe acute respiratory syndrome coronavirus 2 (SARS-CoV-2), was first identified in December 2019 in Wuhan, China, and rapidly spread across the globe [1]. By June 2024, over 775 million individuals worldwide had contracted COVID-19, resulting in more than seven million deaths [2]. Compared to the initial period of the pandemic, morbidity and mortality have significantly decreased over time. This reduction is largely attributed to the adaptation of health systems, widespread vaccination efforts, and the emergence of less virulent virus variants [3]. The clinical manifestations of COVID-19 vary widely, from asymptomatic cases to severe respiratory failure [4]. While 80% of cases exhibit mild to moderate symptoms, 20% progress to severe and critical illness requiring intensive care [5].

COVID-19 is a multisystem disease known to affect various systems beyond the pulmonary system. It has been reported to cause thrombotic complications, myocardial dysfunction, cardiac arrhythmias, and acute kidney injury, among other organ involvements [6-8]. Since the beginning of the pandemic, patients have been evaluated based on their presenting symptoms, vital signs, comorbidities, and laboratory parameters, determining whether they would be managed as outpatients or require hospitalization. Hospitalized patients were closely monitored for vital signs and laboratory parameters, with those needing advanced oxygen therapy or invasive mechanical ventilation being transferred to intensive care units. Predicting the prognosis of COVID-19 is crucial, particularly for cases with a high risk of mortality. Identifying risk factors that influence the course and outcomes of the disease is fundamental in developing strategies to prevent fatal outcomes. Factors such as the patient's age, existing comorbidities, and access to early diagnosis and treatment play a significant role in determining the prognosis [9]. A comprehensive analysis of indicators that can predict the progression of COVID-19 is essential for designing strategic interventions to reduce and manage COVID-19-related mortality rates.

In the early months of the COVID-19 pandemic, the mortality rate was quite high. However, over time, the discovery of vaccines and the emergence of less lethal variants have led to a decrease in mortality rates. The stringent quarantine measures implemented during the initial months likely helped limit the spread of the virus and delay the emergence of new variants. Consequently, due to the strict global quarantine methods in the first six months of the pandemic, the symptoms and clinical characteristics of COVID-19 were similar worldwide, and its progression showed consistency. During a global pandemic, the lack of initial information about the disease often results in high mortality rates until its characteristics, clinical findings, and treatment strategies are established. By evaluating the initial findings of previous pandemics, we can gain insights that may help address future pandemics more effectively.

At this point in our study, we aimed to identify risk factors predicting the progression of COVID-19 to advanced respiratory support (ARS) in patients presenting during the initial months of the pandemic when the virus exhibited its original clinical characteristics. By doing so, we seek to contribute to the early identification of these predictive factors in future pandemics, thereby facilitating timely and appropriate interventions for patients.

## Materials and methods

The first COVID-19 patient in Turkey was reported in March 2020. In this retrospective study, we included patients who were admitted and required hospitalization after being diagnosed with COVID-19 within the first three months of the pandemic (from March to May 2020) in our country. The diagnosis of COVID-19 was confirmed using the reverse transcription polymerase chain reaction (RT-PCR) test. We recorded the age, gender, demographic characteristics, symptom characteristics, comorbidities, admission oxygen saturation, hospitalization duration, in-hospital mortality, and admission routine blood parameters of patients over 18 years old who tested positive for COVID-19 by RT-PCR and did not require advanced respiratory support (ARS) at the time of admission. Advanced respiratory support was defined as the administration of non-invasive ventilation (NIV) or high-flow nasal oxygen (HFNO). The patients requiring invasive mechanical ventilation or ARS at admission were excluded from the study. The included patients were divided into two groups: those who required ARS during follow-up and those who did not. The clinical and laboratory characteristics of these groups were compared. The neutrophil-to-lymphocyte ratio (NLR) and the systemic immune-inflammation response index (SIRI) were calculated from routine hemogram tests. NLR was calculated as absolute neutrophil count/absolute lymphocyte count. The SIRI is calculated as absolute neutrophil count×(absolute monocyte count/absolute lymphocyte count). The study was carried out in accordance with the principles of the Declaration of Helsinki. The study was approved by the Konya Chamber of Commerce (KTO) Karatay University Faculty of Medicine Non-Pharmaceutical and Non-Medical Device Research Ethics Committee (approval number: 2020/017).

Statistical analyses were conducted using the SPSS software (version 25.0 for Windows, IBM SPSS Statistics, Armonk, NY). The Kolmogorov-Smirnov test assessed the distribution of variables. Normally distributed data were presented as mean (±SD), while skewed continuous variables were expressed as medians with interquartile ranges. Categorical variables were represented as percentages. Continuous variables were compared using the independent sample t-test or Mann-Whitney U test, as appropriate. Chi-square tests were used for comparing categorical variables. Baseline variables that were significant (p<0.05) in the univariate analysis were included in a multivariate logistic regression to identify independent predictors of ARS. The results were reported as odds ratios (OR) with 95% confidence intervals (CI) and p values. Receiver operating characteristic (ROC) curve analysis was used to identify the optimal cut-off value of SIRI. A p value of <0.05 was considered statistically significant for all tests.

## Results

The demographic and laboratory values of the 162 patients included in the study are summarized in Table 1. Of the patients, 32.1% required ARS, while 67.9% did not require ARS. Compared to the non-ARS group, statistically significant differences were observed in baseline parameters: higher prevalence of male gender (34 {65.4%} versus 51 {46.4%}, p=0.036), age (61.19±14.76 years versus 46.8±15.33 years, p<0.001), body mass index (BMI) (30.96±4.48 kg/m^2^ versus 27.13±3.43 kg/m^2^, p<0.001), coronary artery disease prevalence (10 {19.2%} versus seven {6.4%}, p=0.026), neutrophil count (4.23 {2.82-5.7}×10^3^/μL versus 3.42 {2.31-4.88}×10^3^/μL, p=0.022), C-reactive protein (CRP) levels (46.8 {25.36-107.1} mg/L versus 21 {7.96-31.25} mg/L, p<0.001), ferritin levels (393 {206.8-794.7} ng/mL versus 81.5 {35-116} ng/mL, p<0.001), D-dimer levels (0.42 {0.2-1.31} mg/L versus 0.21 {0.06-0.37} mg/L, p<0.001), troponin T levels (1.91 {1-3.91} ng/L versus 1.04 {0.89-2.1} ng/L, p=0.002), NLR (3.45 {2.22-4.89} versus 1.98 {1.12-3.03}, p<0.001), SIRI (2.37 {1.87-3.67} versus 1.47 {0.86-1.94}, p<0.001), myalgia prevalence (30 {57.7%} versus 37 {33.6%}, p=0.006), and time from symptom onset to admission (five (5-8) days versus five (4-5) days, p<0.001). On the contrary, lower incidences of sore throat (14 {26.9%} versus 49 {44.5%}, p=0.048) and lower lymphocyte count (1.51 {0.91-1.81}×10^3^/μL versus 1.81 {1.36-2.41}×10^3^/μL, p<0.001) were noted in the ARS group. The hospitalization duration (9.5 {8-12} days versus eight {7-9} days, p<0.001) and mortality rate (five {9.6%} versus zero, p=0.003) were significantly higher in the ARS group.

**Table 1:** Baseline characteristics of the study population ^a^Independent sample t-test ^b^Chi-square test ^c^Mann-Whitney U test ARS, advanced respiratory support; BMI, body mass index; SpO_2_, oxygen saturation; hs, high sensitivity

	All patients (n=162)	ARS group (n=52, 32.1%)	Non-ARS group (n=110, 67.9%)	p value
Age (years)	51.42±16.54	61.19±14.76	46.8±15.33	<0.001^a^
Male gender	85 (52.5%)	34 (65.4%)	51 (46.4%)	0.036^b^
BMI (kg/m^2^)	28.36±4.19	30.96±4.48	27.13±3.43	<0.001^a^
Cough	53 (32.7%)	22 (42.3%)	31 (28.2%)	0.107^b^
Dyspnea	50 (30.9%)	21 (40.4%)	29 (26.4%)	0.105^b^
Sore throat	63 (38.9%)	14 (26.9%)	49 (44.5%)	0.048^b^
Loss of taste and smell	83 (51.2%)	21 (40.4%)	62 (56.4%)	0.057^b^
Myalgia	67 (41.4%)	30 (57.7%)	37 (33.6%)	0.006^b^
Symptom to admission (days)	5 (4-6)	5 (5-8)	5 (4-5)	<0.001^c^
Smoking	65 (40.1%)	23 (44.2%)	42 (38.2%)	0.574^b^
Hypertension	47 (29%)	20 (38.5%)	27 (24.5%)	0.102^b^
Diabetes mellitus	29 (17.9%)	11 (21.2%)	18 (16.4%)	0.601^b^
Coronary artery disease	17 (10.5%)	10 (19.2%)	7 (6.4%)	0.026^b^
Congestive heart failure	12 (7.4%)	6 (11.5%)	6 (5.5%)	0.202^b^
Chronic obstructive pulmonary disease	17 (10.5%)	9 (17.3%)	8 (7.3%)	0.095^b^
Chronic renal disease	6 (3.7%)	4 (7.7%)	2 (1.8%)	0.084^b^
Admission SpO_2_ <92%	45 (27.8%)	17 (32.7%)	28 (25.5%)	0.337^b^
Admission fever	52 (32.1%)	21 (40.4%)	31 (28.2%)	0.120^b^
Hemoglobin (g/dL)	13.56±1.54	13.23±1.82	13.71±1.38	0.096^a^
Leukocytes (×10^3^/μL)	6.15 (4.57-7.92)	6.17 (4.4-7.91)	6.15 (4.79-8.01)	0.795^c^
Neutrophils (×10^3^/μL)	3.68 (2.38-5.21)	4.23 (2.82-5.7)	3.42 (2.31-4.88)	0.022^c^
Lymphocytes (×10^3^/μL)	1.61 (1.22-2.34)	1.51 (0.91-1.81)	1.81 (1.36-2.41)	<0.001^c^
Monocytes (×10^3^/μL)	0.75±0.2	0.79±0.23	0.73±0.18	0.088^a^
Platelets (×10^3^/μL)	226.93±66.97	214.6±74.81	232.76±62.44	0.107^a^
C-reactive protein (mg/L)	24.55 (10.65-48.5)	46.8 (25.36-107.1)	21 (7.96-31.25)	<0.001^c^
Procalcitonin (μg/L)	2 (1-2.76)	2 (1.1-2.1)	2 (1-2.79)	0.528^c^
Ferritin (ng/mL)	106.5 (54.5-224.25)	393 (206.8-794.7)	81.5 (35-116)	<0.001^c^
D-dimer (mg/L)	0.28 (0.1-0.52)	0.42 (0.2-1.31)	0.21 (0.06-0.37)	<0.001^c^
Troponin T (hs) (ng/L)	1.3 (1-2.5)	1.91 (1-3.91)	1.04 (0.89-2.1)	0.002^c^
Creatinine (mg/dL)	0.79 (0.64-0.92)	0.83 (0.71-0.94)	0.74 (0.63-0.9)	0.070^c^
Aspartate aminotransferase (IU/L)	26 (19-34.2)	27.5 (19-34)	24.5 (18-35)	0.323^c^
Alanine aminotransferase (IU/L)	24 (16-36)	21 (13.25-28.75)	24.5 (17-38.25)	0.065^c^
Neutrophil-to-lymphocyte ratio	2.39 (1.52-3.6)	3.45 (2.22-4.89)	1.98 (1.12-3.03)	<0.001^c^
Systemic immune-inflammation response index	1.79 (1.15-2.41)	2.37 (1.87-3.67)	1.47 (0.86-1.94)	<0.001^c^
Hospitalization (days)	8 (8-9)	9.5 (8-12)	8 (7-9)	<0.001^c^
Mortality	5 (3.1%)	5 (9.6%)	-	0.003^b^

Multivariate logistic regression analysis revealed that age (OR, 1.099; 95% CI, 1.027-1.176; p=0.006), elevated CRP levels (OR, 1.026; 95% CI, 1.003-1.049; p=0.024), elevated ferritin levels (OR, 1.026; 95% CI, 1.013-1.039; p<0.001), and SIRI (OR, 7.296; 95% CI, 2.093-25.434; p=0.002) were identified as independent risk factors for the need for ARS in COVID-19 patients (Table 2).

**Table 2:** Independent predictors of mortality in univariate and multivariate analysis OR, odds ratio; CI, confidence interval; hs, high sensitivity

	Univariate analysis			Multivariate analysis		
Variable	OR	95% CI	p value	OR	95% CI	p value
Age (years)	1.065	1.038-1.093	<0.001	1.099	1.027-1.176	0.006
Gender	2.185	1.103-4.327	0.025	6.216	0.917-42.122	0.061
Body mass index (BMI) (kg/m^2^)	1.305	1.174-1.451	<0.001	1.15	0.871-1.52	0.324
Myalgia	2.69	1.366-5.298	0.004	2.728	0.44-16.89	0.281
Symptom-to-admission time (days)	1.578	1.281-1.945	<0.001	1.333	0.713-2.49	0.368
Coronary artery disease	3.503	1.25-9.817	0.017	1.69	0.078-36.846	0.739
Neutrophil count (×10^3^/μL)	1.148	1.022-1.289	0.02	1.117	0.454-2.752	0.81
Lymphocyte count (×10^3^/μL)	0.417	0.251-0.692	0.001	0.594	0.08-4.407	0.610
C-reactive protein (CRP) (mg/L)	1.037	1.023-1.052	<0.001	1.026	1.003-1.049	0.024
Ferritin (ng/mL)	1.022	1.014-1.03	<0.001	1.026	1.013-1.039	<0.001
Troponin (hs) (ng/mL)	1.242	1.084-1.422	0.002	1.032	0.878-1.213	0.704
D-dimer (mg/L)	1.285	1.156-1.428	<0.001	0.155	0.007-3.619	0.246
Neutrophil-to-lymphocyte ratio (NLR)	1.6	1.29-1.984	<0.001	0.546	0.297-1.006	0.052
Systemic immune-inflammation response index (SIRI)	2.247	1.61-3.138	<0.001	7.296	2.093-25.434	0.002

**Figure 1:**
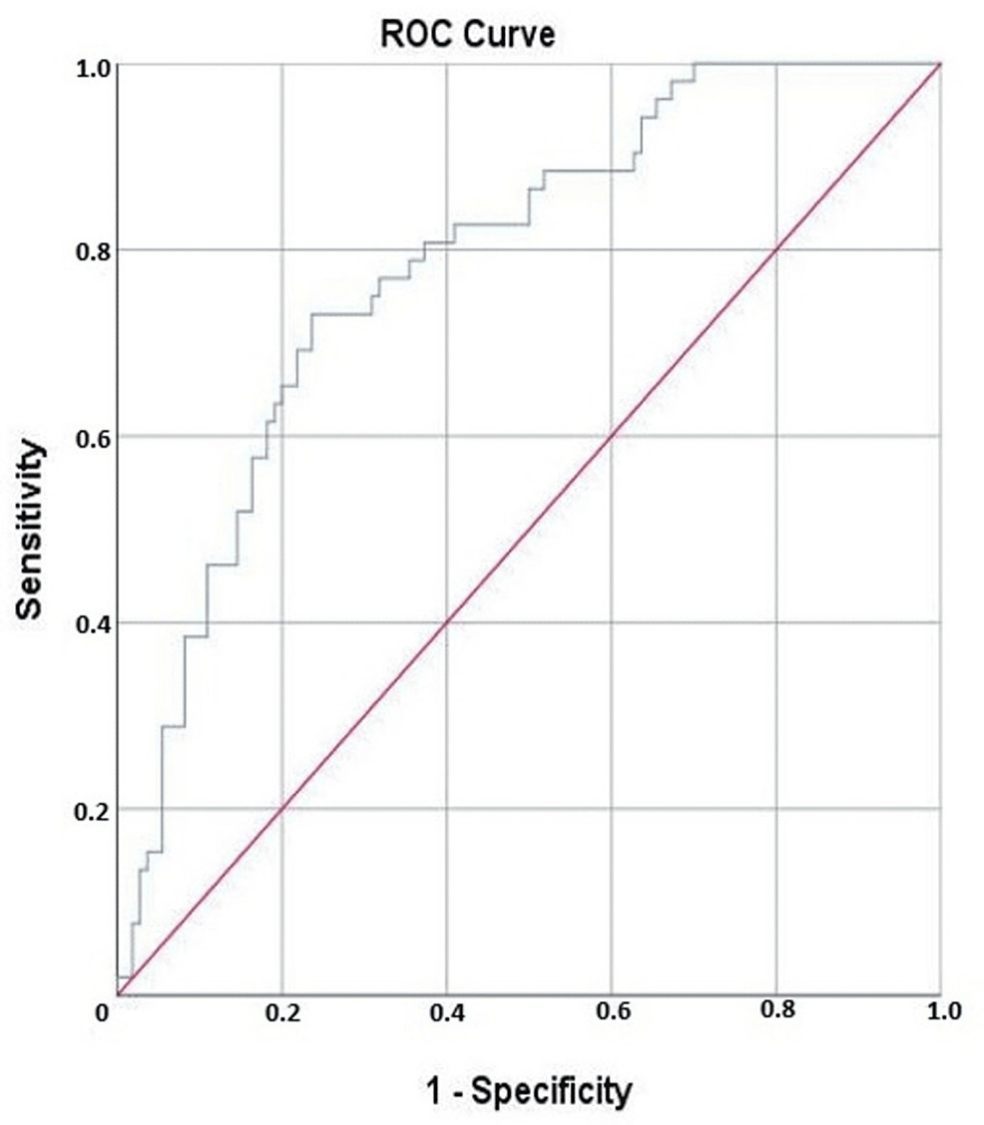
The ROC curve demonstrates the ability of SIRI to predict the need for advanced respiratory support in patients with COVID-19 ROC, receiver operating characteristic; SIRI, systemic immune-inflammation response index; COVID-19, coronavirus disease 2019

In the ROC analysis, the area under the curve (AUC) for the SIRI was calculated to be 0.785 (standard error, 0.037; 95% CI, 0.712−0.857), which was found to be statistically significant (p<0.001). When the cut-off value for SIRI was set at 1.915, it predicted the need for ARS with a sensitivity of 73.1% and a specificity of 72.7% (Figure 1).

## Discussion

The main findings of the current study indicate that increased age, male gender, elevated BMI, the presence of myalgia, coronary artery disease, longer symptom-to-admission interval, and laboratory markers such as neutrophil and lymphocyte counts, CRP, ferritin, troponin, and D-dimer levels, along with NLR and SIRI ratios, were all significantly associated with the need for ARS in COVID-19 patients. More importantly, multivariate analyses identified age, CRP, ferritin, and SIRI as independent predictors of the need for advanced respiratory support in hospitalized COVID-19 patients. Despite the abundance of studies on COVID-19, our study concentrates on the pandemic's first three months. This period was chosen to capture the disease's initial characteristics, free from the influences of variants, mutations, and vaccines. This approach provides a baseline and clear understanding of the disease's original characteristics. Thereby, our findings could serve as a crucial reference during the early stages of any future respiratory pandemic, offering valuable insights before detailed data are collected.

Age is a well-established and significant predictor of poor prognosis in COVID-19 infection, as documented extensively in the literature [10-12]. Our research identified age as a risk factor for the need for ARS among COVID-19 patients. The tissue repair and remodeling responses to lung injuries caused by severe COVID-19 infection are markedly influenced by aging, which are correlated with immunosenescence, heightened inflammatory responses, increased oxidative stress, and diminished cellular repair capacity, thereby exacerbating clinical outcomes and resulting in more extensive lung damage in older individuals [13]. Furthermore, the presence of age-related comorbid conditions may significantly contribute to the adverse progression and heightened mortality of COVID-19 in elderly patients.

In our cohort, CRP and ferritin were identified as predictors of the necessity for advanced respiratory support in COVID-19 patients (p=0.024 and p<0.001, respectively). Both CRP and ferritin are inflammatory markers, and our findings align with other studies that have demonstrated a link between elevated inflammatory markers and poor outcomes in COVID-19 patients [14,15]. Ben Jemaa et al. showed a strong relationship between the severity of COVID-19 and elevated CRP levels [16].

Complete blood count analysis, a widely used and cost-effective blood test, offers rapid and user-friendly results. From this routine test, inflammatory markers such as NLR and SIRI have been employed to predict COVID-19 severity [17,18]. In our study, NLR levels were significantly higher in the patients requiring ARS, but multivariate logistic regression analyses did not retain NLR as a predictor. SIRI emerged as a robust independent predictor for ARS in COVID-19 patients, reflecting the delicate balance between the patient's immune and inflammatory responses. A pronounced inflammatory response can weaken the immune system, making the assessment of systemic inflammation biomarkers crucial for diagnosing and stratifying disease severity. Recently, novel inflammatory ratios derived from routine blood tests have gained traction as key indicators in various situations. Based on ROC curve analyses, Citu et al. [17] established a SIRI cut-off value of 2.2 to predict mortality in COVID-19 patients; similarly, our research identified a SIRI cut-off point of 1.915 to predict ARS in our cohort.

Given the rapid progression to respiratory failure requiring invasive mechanical ventilation and extracorporeal membrane oxygenation and even death in some patients, it is essential to pay close attention to susceptible populations with COVID-19. During the early course of the pandemic, there was significant uncertainty regarding the primary oxygenation strategy. While some advocated for early intubation, others suggested avoiding intubation until the last possible moment. In our clinical practice, we considered the period before intubation, involving patients requiring ARS, as a critical juncture. Thus, we aimed to predict the need for ARS in the patients who did not require it at the time of admission. Methods of respiratory support strategies vary and should be determined by the severity of the illness. Our strategy classified patients into three groups based on their respiratory support needs. The first group included patients in poor general condition at the time of admission who were directly intubated and transferred to intensive care. The second group consisted of patients requiring ARS (non-invasive mechanical ventilation or high-flow oxygen therapy) at admission. The third group comprised patients who did not need ARS at admission but were hospitalized due to comorbidities, age, or general poor condition. Our study focused on this third group, evaluating patients who initially appeared to be in relatively good condition but later developed a need for ARS during follow-up. We identified factors predicting the development of ARS, allowing for earlier interventions and the initiation of more aggressive treatment regimens in high-risk individuals. Moreover, during the COVID-19 pandemic, the significant shortage of medical resources, including mechanical ventilators, underscored the importance of the early identification of patients at risk for ARS during hospitalization.

This study has several limitations. Firstly, its retrospective design may introduce bias. Secondly, the study was conducted during the early months of the COVID-19 pandemic, when clinical practices were still evolving. This may affect the generalizability of our findings. Finally, we did not account for changes in treatment protocols and the impact of vaccines introduced later in the pandemic. For future prospective, multicenter studies are needed to validate our findings.

## Conclusions

This study identifies age, CRP levels, ferritin levels, and SIRI as significant predictors of the need for advanced respiratory support in COVID-19 patients during the early months of the pandemic. These findings highlight the importance of the early identification and monitoring of high-risk patients to facilitate timely interventions. Our results suggest that these predictors could be used to prioritize patients for closer observation and more aggressive treatment strategies, potentially improving outcomes during the initial stages of respiratory pandemics. These insights may facilitate the enhancement of strategies for dealing with future respiratory health issues.
